# Delving Into the Working Mechanism of Prediction in Sentence Comprehension: An ERP Study

**DOI:** 10.3389/fpsyg.2021.608379

**Published:** 2021-02-19

**Authors:** Yunlong Huang, Minghu Jiang, Qian Guo, Yuling Wang

**Affiliations:** ^1^Advanced Innovation Center for Future Education, Beijing Normal University, Beijing, China; ^2^School of Humanities and Social Sciences, Tsinghua University, Beijing, China; ^3^Department of Foreign Languages and Literatures, Tsinghua University, Beijing, China

**Keywords:** prediction, underlying mechanism, memory retrieval, sentence comprehension, ERP

## Abstract

The present study aims to delineate the working mechanism of prediction in sentence comprehension, by disentangling the influence of the facilitated general memory retrieval from the coexistent influence of the predicted language-specific semantic and/or syntactic information for the first time. The results support that prediction might influence the downstream cognitive processing in two aspects: (1) the pre-activated information facilitates the retrieval of a matched input in memory and, (2) the pre-activated information interacts with higher-level semantic/syntactic processing. More importantly, the present findings suggest that these two types of influences seem to occur at different stages of sentence comprehension: the facilitated memory retrieval of the input modulates N400 amplitude and the latency of post-N400 late central-parietal positivity/P600, while the predicted semantic/syntactic information and/or their interactions modulate the amplitude of the late positivity. The present findings would be helpful for interpreting the underlying mechanism of observed effects in prediction studies.

## Introduction

Prediction is an important mechanism in language comprehension (Miller and Isard, 1963; Tulving and Gold, 1963; Forster, 1981; Jackendoff, 2002). It refers to the cognitive process that the brain actively predicts the next input information in advance based on previous contextual information. Many event-related potentials (ERP) studies have found prediction could affect sentence comprehension at different stages (DeLong et al., 2005; Laszlo and Federmeier, 2009; Van Petten and Luka, 2012; Brothers et al., 2015; DeLong and Kutas, 2020; Kuperberg et al., 2020). Specifically, when the input vocabulary matches the predicted information, the amplitude of N400 (a negative-going deflection that peaks around 400 milliseconds post-stimulus onset) will be reduced or even disappear, while violations of predicted information are reported to increase the amplitude of post-N400 positivities (PNP) (e.g., DeLong and Kutas, 2020; Kuperberg et al., 2020). These experimental findings have incrementally improved our understanding of the role of prediction in language comprehension. However, interpreting the specific cognitive mechanisms underlying these observed ERP effects remains to be difficult and inconsistent at present.

General cognitive memory retrieval and higher-level linguistic-specific processing are two major perspectives from which the underlying mechanism of the observed ERP modulations in prediction studies are interpreted. Take N400 as an example, when a reduction of N400 amplitude is observed in a more predictable condition, we have at least two options to interpret the underlying cognitive process: (1) the facilitation of memory retrieval or, (2) the decrease of post-retrieval semantic integration difficulty. The former view suggests that when the new input information is pre-activated in memory as a result of prediction, the retrieval of the pre-activated information will be faster and easier as compared with the not pre-activated input information. Accordingly, less effort is required to access the semantic information of the input, reducing the amplitude of N400. In contrast, the latter view attributes the reduction of N400 amplitude to a decrease of the post-retrieval semantic integration difficulty. It holds that it is easier for the brain to integrate new input information into the previous context when it matches the expected information, which is naturally plausible in the context.

Compared with the disagreement in the N400 time window, the disagreement about the underlying cognitive mechanism interpretation in the P600 time window is more complicated. According to pioneering studies, the reported PNPs include two functionally different positivities: a late frontal positivity and a late parietal positivity/P600 (Van Petten and Luka, 2012; DeLong and Kutas, 2020; Kuperberg et al., 2020). Typically, less predictable implausible inputs (e.g., The tourists visited the pandas at the ***volume****/zoo*) will elicit late parietal positivity/P600, while less predictable but plausible words (e.g., The tourists visited the pandas at the ***website****/zoo*) generally elicit late frontal positivity. Similar to the disagreement in the N400 time window, interpretations of the underlying cognitive mechanism of these observed ERP effects in the P600 time window also include the general memory retrieval account and higher-level linguistic-specific processing account. Different from it, there is more than one type of interpretations about the way general memory retrieval might affect downstream processing in the P600 time window. These interpretations include: (1) an independent role of memory retrieval in the modulation of the observed effects, (2) an interaction between memory retrieval and other coexisting higher-level linguistic-specific processing account to interpret the modulations of the observed effects.

Relatively speaking, the linguistic-specific interpretation of the late positivity changes in the P600 time window is a more popular way to interpret the observed effects. It mainly refers to proposals suggesting that the late positivities elicited by unexpected words reflect extra or prolonged higher-level processing such as integration, adjusting, or reprocessing (DeLong et al., 2014; Brothers et al., 2015; Kuperberg et al., 2020). For example, Kuperberg et al. (2020) propose that the late frontal positivity reflects the updating of the higher-level representation of meaning (which describes the full set of events, actions, and characters being communicated) to accommodate the new unpredicted but plausible information; and the late posterior positivity/P600 reflects the detection of conflict and subsequent reanalysis, repair, or reinterpretation. In addition to the abovementioned accounts, the linguistic-specific interpretations of the late posterior positivity/P600 modulations in prediction studies include another type of explanation, i.e., the interaction between different types of higher-level processing. As several previous studies have proposed, the post-retrieval higher-level processing like semantic integration may interact with syntactic processing at the later stage of comprehension (e.g., Macdonald et al., 1995; Friederici, 2002; Friederici and Weissenborn, 2007; Fedorenko et al., 2012; Traxler, 2014), and the late positivity/P600 at this stage can be significantly modulated by these interactions (e.g., Gunter et al., 2000; Friederici, 2002; Kim and Osterhout, 2005; Friederici and Weissenborn, 2007; Kuperberg, 2007). In prediction studies, the semantic factor (e.g., the semantic integration difficulty) is different between predictable and unpredictable conditions. Therefore, the observed differences in late positivity/P600 in prediction studies could also be interpreted as affected by different linguistic-specific semantic-syntactic interactions.

In contrast to the linguistic-specific interpretations, there are also different proposals suggesting influences of facilitated memory retrieval on downstream comprehension instead of the linguistic-specific processing. As mentioned earlier, these evidences could be briefly categorized into two groups: (1) evidence suggesting a possible independent contribution of memory retrieval to the modulation of late positivities, and (2) the evidence suggesting possible interactions between memory retrieval and higher-level semantic/syntactic processes indexed by late positivities.

### The Possible Contribution of Memory Retrieval to the Modulation of Late Positivities

Several studies have suggested that the differences between late frontal positivities in different conditions might be attributable to memory-retrieval to some extent. For example, Kutas (1993) speculate that the late frontal positivity might reflect inhibition of a predicted-but-not-presented word in memory (see also Ness and Meltzer-Asscher, 2018 for recent discussions). According to inhibition theory (Anderson et al., 1994; Anderson and Spellman, 1995; Verde, 2012), competing information activated in memory will cause potential difficulty for the retrieval and processing of target information. To ensure the processing of target information, an executive control mechanism is required to actively suppress non-targets and facilitate retrieval of the target information, i.e., the retrieval of the target information becomes more difficult in the face of the non-targets. Therefore, this proposal suggests a possible influence of memory retrieval on the cognitive processes in the P600 time window.

Besides the late frontal positivities, several other studies also propose that memory retrieval might contribute to the modulation of the late parietal positivities/ P600 as well (van Petten et al., 1991; Thornhill and Van Petten, 2012; Van Petten and Luka, 2012; Chow et al., 2016). For example, Van Petten and Luka (2012) suggests that memory retrieval might be the possible contributing factor that underlies the variety of P600 effects elicited by difficult and/or anomalous syntactic or certain semantic information. A more recent study also explicitly links prediction to memory accessing mechanism in language processing and suggests that prediction in sentence comprehension could be usefully treated as a memory retrieval problem (Chow et al., 2016). From another perspective, studies focusing on the P600/ late posterior positivity (rather than prediction studies) also suggest an association of the late positivity with memory retrieval, for example, Gouvea et al. (2010) suggests that “variation in the P600 response in particular, may be understood in terms of the prediction, retrieval, and structure building operations needed to create syntactic structure.”

### The Possible Interactions Between Memory Retrieval and Late Higher-Level Processes

Previous studies also report evidence suggesting that the late higher-level grammatical repair and/or reanalysis, which are typically indexed by a central-parietal P600, could possibly be affected by memory retrieval, i.e., memory retrieval might affect the downstream processing through interactions with later higher-level processes.

In prediction studies, Loerts et al. (2013) found that P600 elicited by Dutch grammatical gender mismatch in the high cloze condition (i.e., more predictable condition: “Vera is planting red roses in ^∗^the_*NEU*_ garden_*COM*_ of her parents”) starting significantly earlier as compared to that in the low cloze condition (i.e., less predictable condition: “Vera is knitting a scarf in ^∗^the_*NEU*_ garden_*COM*_ of her parents”), but with no significant amplitude differences. Their results support that higher predictability speeds up the higher-level re-analysis process of the ungrammatical gender mismatch. Similarly, in a study of the underlying mechanism of syntactic P600, Gouvea et al. (2010) finds significant differences in the time latency of P600 effects elicited by different syntactic anomalies. Accordingly, the authors speculate that “the latency of the P600 reflects the time needed to retrieve the elements that participate in a structural relation” (p.183). These studies demonstrated that an easier retrieval in memory facilitates the subsequent higher-level processing in time course.

Besides time latency modulations, there are also experiments reporting a modulation of the amplitude of the syntactic/grammatical P600 in prediction studies. In a study of German gender mismatch in high and low cloze conditions, Gunter et al. (2000) found a longer and significantly larger P600 only in the high cloze condition. According to the authors, longer and larger P600 for gender mismatch in the high cloze condition such as “She travels the_ MASC_ land _*NEUTER*_ …,” as compared to its counterpart in the low cloze condition “She drives the _*MASC*_ land _*NEUTER*_…,” might reflect deeper repair and re-analysis. They suggest that the primary goal in the low cloze condition is to retrieve and extract semantic meaning because it is not predicted (i.e., the cognitive resource allocated for the processing of grammatical gender mismatch is limited), thus a shorter and smaller late positivity; but the meaning is pre-activated in the sentence in high cloze condition, so the cognitive efforts needed to retrieve and extract meaning is saved and the brain only needs to focus on the repair of the gender mismatch (i.e., the facilitated retrieval of semantic meaning freed up the limited cognitive resource for repair and reanalysis). Likewise, Guajardo and Wicha (2014) found that gender mismatch in Spanish elicited a larger positivity relative to gender congruent adjectives only in highly constraining (more predictable) sentences for the LPCa/earlier part of P600 (500–700 ms), and the positivity elicited by gender mismatch began earlier in highly constraining sentences (reached significance at 450–500 ms), compared to weakly constraining sentences (reached significance at 500–550 ms).

In short, these studies suggest that the facilitated memory retrieval of the critical word in the predictable (high constraint/high cloze) condition can possibly alter the amplitude and/or latency of syntactic P600 and seems to interact with later higher-level grammatical violation related processes^1^.

Issues to be addressed:

The mechanism of the facilitated memory retrieval in sentence comprehension is not clear yet, and it, in turn, poses great confusion to the interpretation of the underlying mechanism of the observed ERP effects at different stages in prediction studies. Specifically, it is still difficult to answer whether different modulations of latency and/or amplitude actually come from higher-level language-specific factors like semantic/syntactic processing or more general cognitive influence from memory retrieval, or a combination of both. Therefore, it would be helpful to disentangle the influence of memory retrieval from that of the retrieved semantic/syntactic information on the downstream higher-level processing in sentence comprehension studies, so that we can have a better understanding of the working mechanism of the way prediction affects downstream sentence comprehension.

Challenges at present:

An investigation of the present prediction studies reveals that the present paradigm of studying the influence of prediction on downstream cognitive processes (i.e., with the help of high versus low cloze contexts) are not sufficient enough to differentiate the influences from the general memory retrieval of the input and those from the pre-activated semantic/syntactic information itself. Specifically, if we create predictable and unpredictable conditions through high vs. low context manipulation, the retrieval of the critical word in memory will be significantly facilitated in the predictable condition. But at the same time, the degree of post-retrieval linguistic-specific processing difficulty (e.g., semantic integration reanalysis and/or repair) in the predictable condition will be reduced as well. As a result, it is hard to tell: (1) which one of these two factors actually contributes to the observed modulation of the ERP effects, and/or (2) which one of them interacts with the coexisting late high-level processing (e.g., retrieval-semantic/syntactic interaction vs. semantic-syntactic interaction) and thus modulates the observed ERP effects.

## The Present Study

In order to disentangle the influence of facilitated memory retrieval from that of the pre-activated linguistic-specific semantic/syntactic information on the downstream higher-level processing in sentence comprehension, we aim to control and exclude the confounding influence of the pre-activated plausible semantic/syntactic information on downstream sentence comprehension, but only to manipulate the memory facilitation factor (facilitated memory retrieval vs. non-facilitated memory retrieval) in sentence comprehension in the present study. Specifically, we designed the following modified priming-like paradigm to achieve this goal.

To exclude the confounding influence brought by the plausibility changes of the semantic/syntactic information in the predictable and unpredictable conditions, we plan to use similar incongruous and ungrammatical critical words embedded in the sentential contexts in our study. The incongruous and ungrammatical critical words have no plausible/correct semantic/grammatical information at all to facilitate downstream processing. Thus, using similar incongruous and ungrammatical critical words in different conditions could prevent significant changes in the processing difficulties of semantic integration and/or possible interactions between semantic and syntactic information at later stages. From another perspective, the use of the incongruous and ungrammatical critical words could also elicit N400 and P600, and provides an important basis for investigating the possible influence of facilitated memory retrieval on the cognitive processes in the N400 and P600 time windows.

To achieve the facilitation of the retrieval of the critical incongruous and ungrammatical words in memory, we borrow the idea from the priming studies, i.e., to repeatedly present the critical information and ask the participant to remember the critical information beforehand. The reasons for doing this are: Prediction facilitates memory retrieval of the critical words during comprehension. The facilitation of downstream processing is believed to be achieved by the pre-activation of the predicted information in memory. Similarly, priming also pre-activates information in memory (they might be of differences in terms of passive versus active manner, but the result in terms of pre-activation of the input information and the subsequent facilitation of the retrieval of it in memory should be almost the same, which is the very goal in the present study). Actually, it is also in this sense that prediction, priming and pre-activation are used interchangeably by researchers to refer to the same underlying cognitive processing (e.g., Van Petten and Luka, 2012). For our present purpose, priming has three advantages: (1) it could help us to dissociate the influence of the semantic information and general memory retrieval, which could not be achieved by only manipulating the cloze probabilities of a critical word; (2) repetition priming could be employed to enhance the pre-activation of the input effectively and make the influence last for a longer time, and; (3) the time of repetition could be manually manipulated to reinforce the pre-activation of the input in memory.

We propose two interconnected hypotheses:

(1) If N400 indexes memory retrieval rather than semantic integration, a reduced N400 should be observed in the repetition condition as compared to the non-repetition condition. The reason is that the incongruous semantic information could not be integrated into the context either before or after repetition priming. If there is a reduction of N400 effect, the reduced N400 effect could only be attributed to the facilitated memory retrieval.
(2) Upon observing a reduction of the N400 component which confirms the success of the memory retrieval facilitation, we further make the second hypothesis as follows: if the downstream cognitive processing is influenced by memory retrieval, modulations of the late positivities (in latency and/or amplitude) should be observed. Specifically, we predict an advancement of the time latency in the facilitated memory retrieval condition (repetition condition). And we also predict a difference in amplitude of late positivity, indicating an influence of memory retrieval on the downstream higher-level processes.

## Materials and Methods

### Participants

Thirty Chinese students from Tsinghua University were paid to participate in this experiment. All the participants are right-handed according to the Edinburgh handedness test (Oldfield, 1971). Three were excluded due to excessive artifacts rejection. The ages of the remaining 27 participants (11 males) ranged from 18 to 37 (mean age = 22.15, SD = 3.77). All of them reported a normal or corrected-to-normal vision before the experiment. None of them reported any psychological disorders. All subjects gave written informed consent before participating the experiment.

### Materials

**FIGURE 1:**
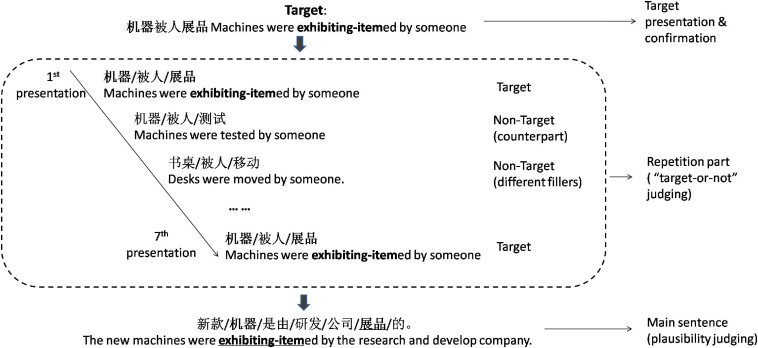
Schematic illustration of the stimulus presentation.

We designed a multiple repetition paradigm to investigate the influence of facilitated memory retrieval of the input itself on the comprehension of sentence. The new paradigm consists of a repetition part and a subsequent main sentence part (see Figure 1). In the repetition part, a shortened core incongruous and ungrammatical structure containing the critical word in the complete critical sentence was extracted for the use of repetition (for more information about the little difference in manipulating the core structure for repetition vs. non-repetition conditions, please see the second paragraph in section “Materials for the Repetition Part”). This shortened core structure was repeated for seven times, together with similar filler expressions in the repetition part. And in the subsequent main sentence part, the complete critical anomalous sentence from which the core structure was extracted was presented immediately.

#### Materials for the Main Part (Main Sentences)

A group of forty correct, passive Chinese sentences were firstly prepared (e.g., “
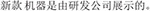
”/ “The new machines were exhibited by the research and develop company”). On the basis of these 40 correct sentences, two groups of incongruous and ungrammatical sentences (40 × 2 = 80) were constructed by replacing the critical transitive verb of each sentence (e.g., “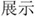; exhibit”) with two nouns (e.g., “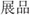; exhibiting-item” and “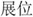; booth”). To prepare for a plausibility validation test, we further created two groups of filler sentences by manipulating congruity and grammaticality of the critical transitive verb of each correct sentence. One group of sentences (40 × 1 = 40) were created by replacing the critical transitive verb with a similar but incongruous transitive verb (e.g., “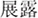; reveal”), while the other group of sentences (40 × 1 = 40) were created by replacing it with an incongruous intransitive verb (e.g., “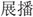; to broadcast”). As a result, there were 200 sentences in five types for plausibility test: 40 correct sentences (congruous transitive verb), 40 incongruous but grammatical sentences (incongruous transitive verb), 40 incongruous and ungrammatical sentences (incongruous intransitive verb), 40 incongruous and ungrammatical sentences (incongruous noun 1), and 40 incongruous and ungrammatical sentences (incongruous noun 2). These sentences were shuffled randomly before the plausibility validation test.

Ratings on a seven-point scale of the plausibility (1 = the most implausible, 7 = the most plausible) of these sentences were acquired from 30 randomly chosen students (who would not participate in the following ERP experiment). The acquired scores of each of these sentences from 30 students were averaged. The data were selected based on the average plausibility scores of the sentences in the two incongruous noun groups. If the plausibility score of a sentence in these incongruous noun groups reaches 3.5, then this sentence and its corresponding sentence counterparts were discarded. As a result, three sets of them were discarded. The scores of the remaining 37 sets of sentences were as follows: the congruous transitive verb group (mean = 6.12, SD = 0.48), the incongruous transitive verb group (mean = 3.26, SD = 0.99), the incongruous intransitive verb group (mean = 2.21, SD = 0.79), the incongruous noun group 1 (mean = 1.78, SD = 0.51), and the incongruous noun group 2 (mean = 1.78, SD = 0.46).

The 37 pairs of incongruous sentences in two incongruous noun groups were selected to be the incongruous critical sentences in the present study. They were very similar in plausibility (Group 1: mean = 1.78, SD = 0.51; Group 2: mean = 1.78, SD = 0.46). The first group was used in the repetition condition, while the second in the non-repetition condition. These two groups of incongruous sentences (37 × 2 = 74 sentences) and the corresponding congruous control sentences (37 sentences, mean = 6.12, SD = 0.48) formed the critical sentence list of the present study.

To create a balanced set of sentences for ERP study, another 129 filler sentences (83 congruous sentences, 46 incongruous sentences) were added. These filler sentences were added to balance the grammaticality and congruity and also to enrich sentence types. As a result, there were 240 complete sentences in total, i.e., 120 congruous sentences (37 critical sentences + 83 fillers) and 120 incongruous sentences (74 critical sentences + 46 fillers).

#### Materials for the Repetition Part

The repetition part was designed as a target judging game. In the target judging game, a short expression was first given as the target. After that, the given target expression was randomly presented together with other non-target filler expressions. Participants were asked to make a judgment about whether each of the expression was the target or not. The short expressions used in this part include three kinds of stimuli: (1) target expression, (2) non-target counterpart expression (very similar to the target expression in structure and key words), and (3) non-target filler expressions (freely coined three-word passive expressions) (see Figure 1).

The target expressions for the repetition condition and the non-repetition condition were constructed differently on the basis of the two groups of incongruous and ungrammatical sentences, since the anomalous sentences should be truly repeated only in the repetition condition. For the repetition condition (group 1), the target expression was directly extracted from the main sentence for the use of repetition (e.g., “
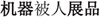
**Machines** were **exhibiting-item**ed by someone” from “
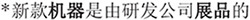
The new **machines** were **exhibiting-item**ed by the research and develop company”). For the non-repetition condition (group 2), one more change was made: the critical word in each expression extracted from the complete sentence was replaced by another word as the target (e.g., “
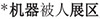
**Machines** were **exhibition-area**ed by someone” rather than “
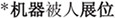
**Machines** were **booth**ed by someone,” which was directly extracted from “
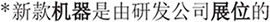
The new machines were **booth**ed by the research and develop company”), see Table 1a for detail.

**TABLE 1:** Example of stimuli in the main part and game part (the incorrect expressions were marked by a preceding “*”).

(a) Example of stimuli in the main part.		

	Preceding Target	Main Stimuli
**Control**		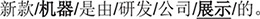
		The new **machines** were **exhibited** by the research and develop company.
**Repetition condition**	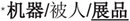	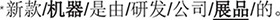
	* M**achines** were **exhibiting-item**ed by someone	* The new **machines** were **exhibiting-item**ed by the research and develop company.
**Non-repetition condition**	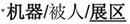	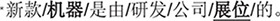
	* M**achines** were **exhibiting-area**ed by someone	* The new **machines** were **booth**ed by the research and develop company.

**(b) Example of stimuli in the preceding game part.**		

	**Target Expressions**	**Non-target Counterpart Expressions**

**Critical Sentence Group 1 (Incorrect Target)**	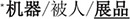	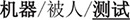
	* M**achines** were **exhibiting-item**ed by someone	M**achines** were **test**ed by someone
**Critical Sentence Group 2 (Incorrect Target)**	^*^ 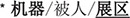	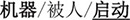
	* M**achines** were **exhibiting-area**ed by someone	M**achines** were **start**ed by someone
**Filler Sentence Group (Correct Target)**	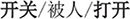	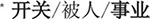
	**Switches** were **turned on** by someone	* Switches were **career**ed by someone

The non-target counterpart expressions were coined based on the targets selected for each group. For each target, we coined a corresponding non-target counterpart in a way that the grammaticality and congruity of the coined non-target was kept contrary to that of the target (i.e., for each ungrammatical and incongruous target, a grammatical and congruous non-target counterpart was coined). As a result, the non-target counterpart expression shared the same patient (the first word) and the same passive structure with the target expression (e.g., “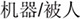/…, Machines were …by someone”), see Table 1b for detail.

The non-target filler expressions were freely coined three-word passive expressions with keywords different from those in both target expressions and non-target counterpart expressions. The non-target filler expression shared only the same passive structure with the target expression (e.g., “…
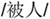
…, …were…by someone). They were put into an independent list for the use of all target-judging games in the study.

Different target expressions were used for matching sentences in the repetition and non-repetition conditions. The non-target counterpart expressions were created for the purpose of grammaticality and congruity balance. The non-target filler expressions were used to avoid monotonous feeling during the target-judging game.

#### Organizing Method of Materials

The target expression was firstly given and presented on the screen before the repetition part starts, waiting for the participants’ confirmation and memorization of the target (see Figure 1). For the repetition condition, the target was the short expression directly extracted from the corresponding main sentence, while for the non-repetition condition, the target expression was different from the core structure extracted from the subsequent main sentence.

The target and its non-target counterpart were each repeated for seven times in a random order in the target-judging game part. Three other fillers were randomly inserted into the repetition part to prevent monotony. Participants were required to judge whether each of the short expressions they saw on the screen was the same as the previously given target. After the repetition part, the corresponding complete critical sentence was presented immediately. The participants were asked to make a plausibility judgment of this complete sentence.

All the critical sentences in repetition, non-repetition conditions, and a subset of the congruous filler sentences (37 filler sentences) were selected to be preceded by the target-judging game. According to the main focus of the present study (i.e., the differences between the critical sentences in the repetition and non-repetition conditions) and the time cost limitation of the experiment, the critical sentences in the control condition were not preceded by the target-judging game part. For future studies that need to include more experimental conditions, further adjustments to the present paradigm might be considered as well, e.g., to reduce the repetition times. All the stimuli in the present study were divided into four blocks. Participants took a 10-minute break between blocks. Before the formal test, each participant practiced with five sentences. The experiment, including electrodes preparation, lasted about 3 h.

### Procedures

Participants were seated in a comfortable chair in a room with dim light. They were instructed to try their best to keep their eyes focused on the middle of the screen and avoid extra body movements but be relaxed during the experiment. All the participants were explicitly told that each incorrect sentence would be followed by a target-judging game part and each correct sentence would be followed by another correct sentence. All the stimuli were presented word-by-word on a LCD computer screen one meter away from the participants. Participants were asked to respond by pressing one of the two buttons, which represent correct and incorrect, respectively, on an Xbox handle with their left or right index fingers while making their judgments. Either of the two buttons could be pressed while they were confirming a given target expression.

The stimuli in the main part were set to dark green against silver gray background. Each sentence began with a “+” sign lasting for 500 ms, and each word was presented for 400 ms followed by a 400 ms blank. At the end of each sentence, three question marks “???” were presented and remained on the screen until a response was given.

The stimuli in the game part were set to navy blue against silver gray background. The target expression was firstly presented on the screen, waiting for confirmation from the participants. In the repetition part, after the confirmation of the target, each short expression began with a “+” sign lasting for 300 ms, and each word in the expression was presented for 300 ms with no intervals between words but followed by a 500 ms blank after the final word, and subsequently, a questioning cue “..” waiting for the participants’ responses was presented and remained on the screen until an answer was received.

### Data Recording

EEG waves were recorded from 62 Ag/AgCl electrodes in an elastic cap (Easycap, Brain Products GmbH), which correspond to the following sites of the international 10–20 system: Fp1, Fpz, Fp2, AF7, AF3, AFz, AF4, AF8, F7, F5, F3, F1, Fz, F2, F4, F6, F8, FT7, FC5, FC3, FC1, FC2, FC4, FC8, FT8, T7, C5, C3, C1, Cz, C2, C4, C6, T8, TP9, TP7, CP5, CP3, CP1, CPz, CP2, CP4, CP6, TP8, TP10, P7, P5, P3, P1, Pz, P2, P4, P6, P8, PO7, PO3, POz, PO4, PO8, O1, Oz, and O2. The vertical electrooculogram (VEOG) was recorded from electrodes below the left eye, and the horizontal electrooculogram (HEOG) from electrodes at the outer canthus of the right eye. Electrode impedances were maintained below 5 kΩ. The EEG signal was amplified by the BrainAmp DC amplifier system (Brain Products GmbH) with a bandpass from 0.01 to 100 Hz and was continuously sampled at 500 Hz.

### Data Analysis

Recordings were re-referenced to the averaged value of left and right mastoids offline. ERP epochs of interest were time-locked to the critical words of the main sentences (presented after the repetition game). The epoch of interest spans from −200 to 900 ms relative to the onset of the critical word. Pre-stimulus 200 ms data was averaged and used as baseline. Analysis was focused on the 800 ms epoch after the onset of the critical word. All the data were filtered with a 30 Hz low-pass filter (A 15 Hz low-pass filter was applied only for the purpose of figure plotting). Trials contaminated by eye blinks or excessive movement (mean voltage exceeding ± 100 μv) were rejected. The ocular artifacts were detected firstly by algorithms implemented in Brain Vision Analyzer 2.0, and then rechecked manually trial by trial. Data of three participants were excluded because the overall rejection rate of the EEG data exceeded 25%. The overall rejection rate of the twenty-seven participants kept for calculation was 6.20% (control condition: 4.60%, repetition condition: 7.31%, non-repetition condition: 6.71%). The overall average number of trials kept for calculation was 34.70 trials (control condition: 35.30 trials, repetition condition: 34.30 trials, and non-repetition condition: 34.52 trials). ERPs were averaged within each experimental condition (control, repeated, unrepeated) for each subject at each electrode site.

The time window was from 300 to 500 ms for the N400 effect and from 500 to 800 ms for the overall P600 effect. Repeated measures analyses of variance (ANOVA) were conducted based on the mean amplitudes in each time window. Factors including CONDITIONS (3 levels: control, repeated violation, and unrepeated violation), LATERALITY (3 levels: left, medial, and right), and ANTERIORITY (3 levels: frontal, central, and parietal). Accordingly, electrodes on the scalp were divided into nine regions of interest (ROIs), and the average amplitude of the electrodes was compared across different ROIs: left-frontal (F7, F5, F3, FT7, FC5, FC3), left-central (T7, C5, C3, TP7, CP5, CP3), left-posterior (P7, P5, P3, PO7, PO3), medial-frontal (F1, Fz, F2, FC1, FCz, FC2), medial-central (C1, Cz, C2, CP1, CPz, CP2), medial-posterior (P1, Pz, P2, O1, POz, O2), right-frontal (F4, F6, F8, FC4, FC6, FT8), right-central (C4, C6, T8, CP4, CP6, TP8), and right-posterior (P4, P6, P8, PO4, PO8). Following previous studies (e.g., Kuperberg et al., 2007; Jiang and Zhou, 2012), we applied repeated measures ANOVAs in all the separate comparisons to detect subtle differences between conditions or in different ROIs. The Greenhouse-Geisser correction was applied when effects with more than one degree of freedom were evaluated in all the overall and separate statistical comparisons. In separate comparisons, Bonferroni correction was used to prevent Type 1 error.

In order to statistically compare the latency differences between conditions, we applied an automatic peak detection procedure provided by Brain Vision Analyzer 2.0 to the ERPs of each participant in the 500–800 ms time interval at all the electrodes in central and parietal ROIs, in which the great majority of P600 effects were usually reported. The latency values of the most prominent peak in each of the conditions were exported and compared statistically by repeated measures analyses of variance (ANOVA). For more detailed comparisons between the elicited P600 effects in the two anomalous sentence groups (repeated vs. unrepeated), we followed Liotti et al. (2000), and further conducted consecutive statistical analysis of each 100 ms time window from 500 to 800 ms for possible, subtler differences. The time windows for all the three conditions were: 500–600, 600–700, and 700–800 ms. These two types statistical results are discussed together.

## Results

### Behavior Results

The overall average accuracy is 96.62% (SD = 1.81) for the complete sentence plausibility judging task in the main sentence part and 98.01% (SD = 1.31) for the target-judging task in the repetition part. The results suggest that all the participants took the experiment seriously.

### ERP Data

**FIGURE 2:**
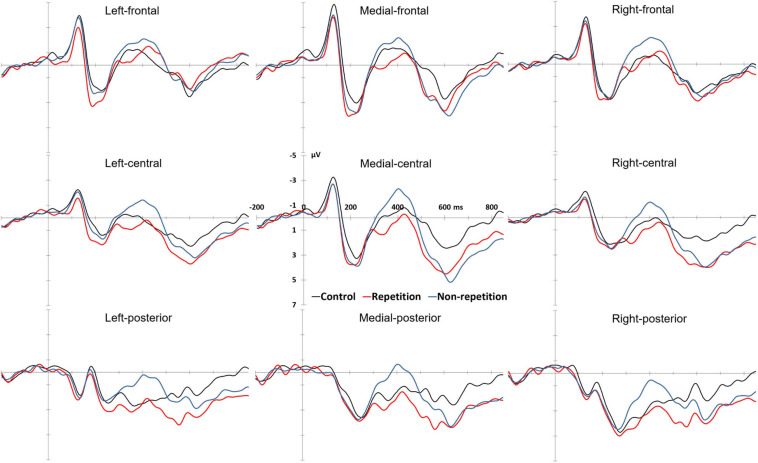
ERPs at all the ROIs (black: control condition, red: repetition condition, blue: non-repetition condition).

**FIGURE 3:**
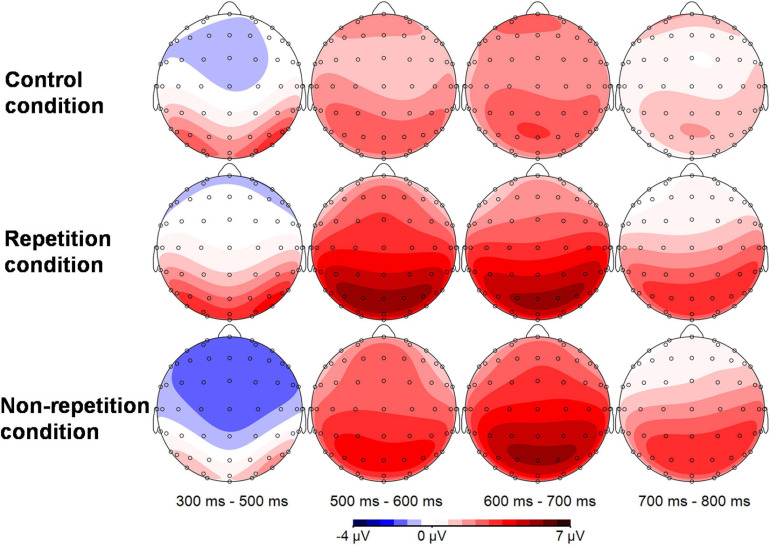
Scalp topographic distributions of different conditions in different time windows (1st column: scalp distribution of different conditions in the N400 time window, 2nd–4th column: scalp distribution of different conditions in the P600 time window).

Grand average ERP waveforms and topographic distribution of the ERP effects are shown in Figures 2, 3, respectively. The descriptive statistics and overall ANOVA results for N400 and P600 effects are presented in Table 2.

**TABLE 2:** The descriptive statistics and overall ANOVA results for N400 and P600 effects.

		Mean	SE	95% CI-L	95% CI-U	d.f.	*F*	*p*
**N400 amplitude** (μV)	Control	0.635	0.323	–0.028	1.299	2,52	12.594	< 0.001
	Repetition	1.134	0.438	0.233	2.034			
	Non-Repetition	–0.423	0.378	–1.199	0.354			
**P600 amplitude** (μV)	Control	1.264	0.423	0.395	2.133	2,52	5.914	= 0.007
	Repetition	2.511	0.409	1.670	3.351			
	Non-Repetition	2.296	0.465	1.341	3.252			
**P600 latency** (ms)	Repetition	597.003	7.456	581.677	612.330	1,26	24.312	< 0.001
	Non-Repetition	626.841	6.689	613.092	640.590			

*SE, standard error; 95% CI-L, the lower bound of 95% confidence interval; 95% CI-U, the upper bound of 95% confidence interval; d.f., degree of freedom.*

#### N400 Amplitude

For the 300–500 ms time window, an overall repeated measures ANOVA (condition × anteriority × laterality) reveals a main effect across conditions, *F*(2,52) = 12.594, *p* < 0.001, with more negative deflections in the unrepeated condition. Separate repeated measures ANOVA comparisons (condition × anteriority × laterality) were conducted to detect whether there were significant differences between each condition pairs. To prevent Type 1 error, Bonferroni correction was used which resulted in a corrected alpha of 0.0167 (0.05/3 = 0.0167). Results demonstrate that the difference between the repeated anomalous conditions and the control condition is not significant, *F*(1,26) = 1.728, *p* = 0.20, but a significant difference between the unrepeated anomalous and the control condition *F*(1,26) = 23.124, *p* < 0.001, with more negative deflections in the unrepeated condition. A comparison between the repeated and unrepeated conditions also reveals a significant difference *F*(1,26) = 22.267, *p* < 0.001, with the anomalous sentences in the repeated condition showing a significant reduction in the N400 response.

The overall repeated measures ANOVA reveals no significant interaction between condition and anteriority, *F*(4,104) = 2.701, *p* = 0.077, and no interaction between condition and laterality, *F* < 1.

#### P600 Amplitude

For the traditional P600 time window (500–800 ms), an overall repeated measures ANOVA (condition × anteriority × laterality) comparison of the mean amplitudes from 500 to 800 ms reveals a main effect across conditions, *F*(2,52) = 5.914, *p* = 0.007, with more positive deflections observed in both repeated and unrepeated anomalous conditions. Subsequent separate repeated measures ANOVA comparisons (condition × anteriority × laterality) were conducted to detect whether there were significant differences between each condition pairs. To prevent Type 1 error, Bonferroni correction was used which resulted in a corrected alpha of 0.0167 (0.05/3 = 0.0167). Results reveal that the repeated anomalous sentences elicited significantly more positive deflections than the sentences in the control condition, *F*(1,26) = 7.419, *p* = 0.011. The unrepeated anomalous sentences also elicited significant positivity, as compared with sentences in the control condition, *F*(1,26) = 8.010, *p* = 0.009. However, separate comparison between the repeated and unrepeated anomalous conditions reveals no significant effect, *F*(1,26) < 1.

The overall repeated measures ANOVA reveals an interaction between condition and anteriority as well, *F*(4,104) = 5.389, *p* = 0.007. Subsequent repeated measures ANOVA (condition × laterality) at each level of anteriority reveals no significant effect of condition in the anterior ROIs, *F* < 1. But there are significant effects of condition in the both the central ROIs, *F*(2,52) = 10.100, *p* < 0.001 and the parietal ROIs, *F*(2,52) = 13.534, *p* < 0.001.

The interaction between condition and laterality does not reach significant, *F*(4,104) = 2.330, *p* = 0.090.

#### P600 Latency

Since the P600 effects exist only in the repetition and non-repetition conditions, we conducted a direct repeated measures ANOVA to the means of the P600 latency in the repetition and non-repetition conditions in all the six central and parietal ROIs where the P600 effects reached significant. The overall repeated measures ANOVA (condition × anteriority × laterality) reveals a significantly earlier occurrence of the P600 effect in the repeated condition, comparing with the unrepeated conditions (mean latency: 597.00 vs. 626.84 ms), *F*(1,26) = 24.312, *p* < 0.001. Based on the mean latency values, the most prominent P600 effect in the repeated condition demonstrated an average advancement of 29.838 ms in occurrence than that in the unrepeated condition. In order to further investigate the latency difference effect, we followed Liotti et al. (2000) and conducted more detailed consecutive comparisons of the P600 effects between the repeated and unrepeated anomalous sentence groups in consecutive time windows, with a length of 100 ms. As a result, we found a significant difference between the repeated and unrepeated conditions in the 500–600 ms time window, *F*(1,26) = 5.688, *p* = 0.025, with the repeated condition a more positive value than the unrepeated condition. But there are no significant differences in both of the 600–700 or 700–800 ms time windows, *Fs* < 1. Direct observation of the waveform in the first time window demonstrated systematic differences in latency rather than in magnitude. Therefore, we propose that the difference in amplitude in the 500–600 ms actually confirms the difference in latency between two conditions.

## Discussion

The present study investigates the independent influence of memory retrieval of the input information on the downstream processing in sentence comprehension, aiming to dissociate the intertwining influences of memory retrieval and higher-level semantic/syntactic factors in prediction studies and provide a better understanding of the working mechanism of prediction during language comprehension. Incongruous and ungrammatical critical words are employed to dissociate the intertwining influence of the facilitated memory retrieval of the critical word from the possible influences of the pre-activated semantic/syntactic information. Repetition priming is borrowed to pre-activate the critical word and facilitate the retrieval of it in memory. The incongruous and ungrammatical information is required to be memorized by the participants first and then repeated for seven times in a following-up target judging game, in order to reinforce the memorization of the critical word and greatly facilitate the retrieval of the incongruous input word. The recorded ERPs of an immediate follow-up semantically enriched sentence containing the repeated incongruous and ungrammatical critical word (the repetition condition) is compared with those of a sentence not containing the repeated word but a different incongruous and ungrammatical critical word (the non-repetition condition). The results in the non-repetition condition demonstrate a biphasic N400 and P600 pattern, consisting with several previous Chinese studies (Ye et al., 2006; Zhang et al., 2013; Yang et al., 2015), while the N400 effect totally disappears in the repetition condition, which agrees with previous findings (Misra and Holcomb, 2003; Kutas and Federmeier, 2011; Stroberg et al., 2017). We discuss these findings and their implications in detail in the following section.

### The Influence of the Facilitated Memory Retrieval on the N400 Effect

An attenuation of the N400 effect in the repetition condition (more predictable) has been revealed by the present study. This finding demonstrates that the N400 effects elicited by semantically incongruous words could be totally eliminated by repetition (which does not change the incongruity of the critical word). Therefore, we suggest that the eliminated N400 effects manifest the continuous facilitating effects of repetition priming on memory retrieval of the input, rather than semantic integration difficulty caused by the incongruous input. Accordingly, we suggest that the successfully facilitated memory retrieval of the critical word provides us with a basis to investigate the influence of facilitated memory retrieval of the input on the downstream higher-level processing in the P600 time window.

### The Influence of the Facilitated Memory Retrieval on the Downstream Higher-Level Processing

A significant advancement of the time latency of the post-N400 late central-parietal positivity/P600 is found in the repetition condition (more predictable) as compared with the non-repetition condition. The advanced time latency agrees with previous studies of syntactic P600 studies (Vos et al., 2001; Gouvea et al., 2010) and studies of prediction (Loerts et al., 2013; Guajardo and Wicha, 2014). We speculate the differences in latency between the repetition condition and the non-repetition condition might reflect the decrease of time cost caused by the pre-activated input information. Therefore, we suggest that difficulties encountered in retrieving unpredicted anomalous information seem to be reflected by the latency of P600.

In terms of amplitude modulations, both of the incongruous and ungrammatical conditions (repetition and non-repetition) elicit central-parietal P600 effects. The results agree with several previous studies (e.g., Ye et al., 2006; Zhang et al., 2013; Yang et al., 2015; Fromont et al., 2020). Although there is a difference in terms of memory retrieval facilitation between repetition and non-repetition conditions, the amplitudes of P600s in these two anomalous conditions were not statistically different from each other (based on an overall averaged data comparison from 500 to 800 ms time window). Note that separate subtle comparisons based on a consecutive 100 ms time window in the 500–800 ms time window reveal a significant difference in 500–600 ms interval in amplitude between the repetition and non-repetition conditions, with a more positive amplitude in the repetition condition. However, a closer inspection of the waveform in the 500–600 ms interval suggests that the differences between these two anomalous conditions is more likely caused by the latency difference than by the amplitude (see Figure 2).

As a result, the facilitated memory retrieval of the input caused by pre-activation in the repetition condition does not increase the amplitude of the P600 effect in the present study. The present results do not agree with those in several prediction studies in which the amplitude of the P600 is increased as a whole (Gunter et al., 2000) or as a part (Guajardo and Wicha, 2014) in more predictable conditions, but agrees with (Loerts et al., 2013; Freunberger and Roehm, 2016) in which the pre-activated information in the more predictable condition does not affect the amplitude of P600. Therefore, the present results could not provide evidence for associating the central-parietal P600 amplitude with the memory retrieval of the input information itself. We speculate that the amplitude of central-parietal positivity might reflect language-specific higher-level cognitive processing other than the relatively more general memory retrieval of the input word. Accordingly, we suggest the P600 amplitude increases in Guajardo and Gunter might reflect the language-specific differences such as the task or language differences and/or complex semantic-syntactic interactions between the predicted semantic/syntactic information and the ungrammaticality of the input. In short, the present results suggest that the amplitude change of central-parietal P600 might not reflect the difficulty of the memory retrieval of input information and is more likely to be attributable to language-specific higher-level processing such as reprocessing or semantic-syntactic interaction.

In addition, no evidence for frontal late positivities in the P600 time window is found in the present study. Frontal late positivity has been associated with the disconfirmation of the predicted information by previous studies (e.g., Federmeier et al., 2010; Thornhill and Van Petten, 2012; Van Petten and Luka, 2012; Kuperberg et al., 2020). Accordingly, we propose the absence of frontal late positivity in the present study might reflect that there is either no extra disconfirmation-related processes initiated due to the incongruous nature of the pre-activated information or the cognitive resources required for disconfirming pre-activated incongruous information in memory is different from that required for disconfirming pre-activated congruous information which typically elicits a frontal late positivity, i.e., the disconfirmation of the incongruous information pre-activated in memory might cost very little cognitive efforts and therefore no frontal late positivity is elicited.

Taken together, the present study successfully facilitated the memory retrieval of the input word, which is indexed by the disappearance of N400 in the repetition condition. On this basis, possible modulations of the late positivities in the post-N400 time window are examined. Typical central-parietal syntactic P600 effects are elicited by the anomalous critical words in both of the repetition and non-repetition ungrammatical conditions, but no frontal late positivity is found in the present study. The present results demonstrate that facilitated memory retrieval of the input word changes the latency of the late higher-level processing indexed by central-parietal P600, but memory retrieval of the input word does not significantly affect the amplitude of syntactic P600. The differences between the amplitude of syntactic P600 are more likely to be triggered by the pre-activated semantic/syntactic contents.

### The Implications for the Working Mechanism of Prediction in Sentence Comprehension

All of the above discussions about the influence of the facilitated memory retrieval of the input information on the downstream processing shed light on inferring the working mechanism of prediction in language comprehension. Prediction pre-activates related information based on the prior context and thus the retrieval of the input information is greatly facilitated or the brain even does not need to retrieve the input information anymore; from another perspective, the predicted semantic/syntactic factors might also facilitate downstream semantic integration and/or cause complex interactions with syntactic factors. Due to disagreements about the underlying mechanism(s) of ERP components and the above-mentioned multiple possibilities for interpreting the contributor of N400 and post-N400 late positivity changes in prediction studies, uncertainties about the underlying working mechanism of prediction in sentence comprehension arise and remain to be resolved. Previous prediction studies found that the more predictable information modulates the amplitudes of both N400 and post-N400 late positivities (Van Petten and Luka, 2012; DeLong et al., 2014; Brothers et al., 2015; DeLong and Kutas, 2020; Kuperberg et al., 2020) and also the latency of P600 (Loerts et al., 2013; Guajardo and Wicha, 2014), but in contrast, the present study focuses only on the influence of memory retrieval of the input information on the downstream processing reveals the facilitated memory retrieval only modulate the N400 amplitude and the latency of the central-parietal late positivity/P600, but not the amplitude of the central-parietal late positivity/P600. Taken together, the present results suggest that the complex influence of prediction on the downstream processing of sentence comprehension at least comes from two sources: memory retrieval and the predicted semantic/syntactic information, and these two sources seem to influence the downstream processing at different stages independently. Specifically, we propose that the facilitation of memory retrieval of the input word caused by the pre-activated information in predicted condition affects the amplitude of the N400 effect and the latency of the post-N400 late positivity, and the predicted contents, such as semantic and/or syntactic information, affects the amplitude of the post-N400 late positivity. For example, we propose, based on the present findings, that the latency changes in Loerts et al. (2013) and Guajardo and Wicha (2014) is more likely to be brought by the facilitation of the retrieval of the input information in memory, but the amplitude changes in Gunter et al. (2000) and Guajardo and Wicha (2014) might be caused by the predicted contents, such as semantic or syntactic information, and/or their interactions.

## Conclusion

Prediction pre-activates related information based on prior contextual support and affects the downstream processing. However, the pre-activated information could possibly influence sentence comprehension in two ways: facilitating general memory retrieval of the pre-activated information and reducing the difficulties in later higher-level processing, such as semantic integration or reprocessing. These two sources of influence entangle with each other and caused complex influences on downstream comprehension. The present study employs incongruous and ungrammatical word as stimuli and repeatedly present them for seven times to facilitate the memory retrieval of the input word. By doing so, we achieved facilitation of the memory retrieval of the critical input word and at the same time excluded the confounding co-existing semantic/syntactic influences while pre-activating a congruous critical word, which is difficult to control for the present context manipulation paradigm in prediction studies. By independently investigating influences of the facilitated general memory retrieval of input information on downstream comprehension, the present study provides a new perspective to delineate the complex influences of the pre-activated information in prediction studies. Based on the present findings, we propose that the modulations of N400 amplitude in prediction studies come from the facilitated memory retrieval of the input information which is fully/partially pre-activated by prediction, the latency advancement in the more predictable conditions in prediction studies come from the shortened time span needed to retrieve the input information, and the modulations of the amplitude of central-parietal positivity/P600 in many prediction studies might actually come from interactions between the predicted semantic/syntactic contents and language-specific higher-level integration and/or reprocessing.

## Data Availability Statement

The raw data supporting the conclusion of this article will be made available by the authors, without undue reservation.

## Ethics Statement

The studies involving human participants were reviewed and approved by Center for Psychology and Cognitive Science, Tsinghua University. The patients/participants provided their written informed consent to participate in this study.

## Author Contributions

YH designed and conducted the experiment and wrote the manuscript. MJ discussed the design and analyzed the results. QG analyzed the results and rewrites parts of the manuscript. YW conducted the experiment and discussed the results. All authors contributed to the article and approved the submitted version.

## Conflict of Interest

The authors declare that the research was conducted in the absence of any commercial or financial relationships that could be construed as a potential conflict of interest.

## Footnotes

1 Note that interactions between the prediction facilitation and grammatical violation can be different depending on the types of grammatical violation, according to a recent study. In a pioneering study of subject-verb violation processing in predictable and unpredictable conditions, Freunberger and Roehm (2016) reports no significant latency or amplitude differences of P600 effects, but only significant different N400 effects between predictable and unpredictable conditions. From this perspective, further studies on the influence of prediction facilitation on more types of grammatical violations are necessary.
